# Schlag auf Schlag – Bericht über feuerwerksbedingte Knalltraumata zum Jahreswechsel 2021/2022

**DOI:** 10.1007/s00106-022-01259-6

**Published:** 2023-01-05

**Authors:** Veronika Flockerzi, Bernhard Schick, die German Acoustic Fireworks-associated Traumata Study (GAFATS) Group, Stephan Hackenberg, Justus Ilgner, Gerhard Hesse, Peter Jecker, Tanja Drews, Herbert Eichwald, Steffen Dommerich, Andreas O. H. Gerstner, Stephanie Hoppe, Jörg Ebmeyer, Jan Peter Thomas, Friedemann Papst, Joachim Hornung, Stephan Lang, Andreas Knopf, Philipp Dost, Christoph Arens, Christian Wrobel, Jörg Langer, Adrian Münscher, Alexandra Gliese, Thomas Lenarz, Olcay Cem Bulut, Matti Hein, Johanna Inhestern, Veronika Flockerzi, Bernhard Schick, Alessandro Bozzato, Philippe Federspil, Petra Ambrosch, Sandra Schmidt, O. Ebeling, Efastathios Papatsoutsos, Claudia Scherl, Haralampos Gouveris, Sandra Schmidt, Boris A. Stuck, Benedikt Hofauer, Base Al Kadah, Klaus Bumm, Martin C. Jäckel, Gregor Hilger, Birgit Muschal, Sven Becker, Theo Evers, Alessandro Bozzato

**Affiliations:** Klinik für Hals‑, Nasen- und Ohrenheilkunde, Universitätsklinikum des Saarlandes, 66421 Homburg, Deutschland

**Keywords:** Umwelt, Feuerwerk, Lärminduzierter Hörverlust, Tinnitus, Epidemiologie, Environment, Fireworks, Noise-induced hearing loss, Tinnitus, Epidemiology

## Abstract

**Hintergrund:**

Ziel war die Erhebung, wie häufig und welche Art von feuerwerksbedingten Knalltraumata in Deutschland um Silvester 2021 trotz Verkaufsverbots für Feuerwerkskörper aufgrund der COVID-19-Pandemie auftraten.

**Material und Methoden:**

Der Erhebungszeitraum umfasste 7 Tage (28. Dezember 2021 bis 03. Januar 2022). In einem Fragebogen wurden Datum, Art und Behandlung des Traumas, Geschlecht und Alter der Patienten erhoben sowie abgefragt, ob das Trauma beim Zünden oder Betrachten von Feuerwerk auftrat. Die Hörbeeinträchtigung wurde nach der Einteilung der Weltgesundheitsorganisation (WHO-Grade 0–4) klassifiziert und begleitender Tinnitus, Schwindel oder andere Verletzungen erfasst. Der Fragebogen wurde an die Hals-Nasen-Ohren-Abteilungen von 171 Krankenhäusern in Deutschland verschickt.

**Ergebnisse:**

Von 37 HNO-Abteilungen meldeten 16 keine und 21 Abteilungen meldeten 50 Patienten mit feuerwerksbedingtem Knalltrauma. Das Durchschnittsalter betrug 29 ± 16 Jahre. Die Mehrzahl der Patienten war männlich (41 von 50). Es stellten sich 22 Patienten ohne und 28 mit Hörverlust vor, 32 berichteten über Tinnitus und 3 über Schwindel. Beim Zünden verletzten sich 20 Patienten und 30 als Bystander. Die Hörminderung wurde in 14 × WHO-Grad 0, 5 × WHO-Grad 1, 4 × WHO-Grad 2, 2 × WHO-Grad 3 und 3 × WHO-Grad 4 eingestuft. Stationär wurden 8 Patienten behandelt, 11 erlitten gleichzeitig Brandverletzungen.

**Schlussfolgerung:**

Trotz des Verkaufsverbots gab es zum Jahreswechsel 2021/2022 einige Knalltraumata durch Feuerwerkskörper. Einige davon führten zu Krankenhausaufenthalten, eine deutlich höhere Dunkelziffer ist zu vermuten. Diese Studie kann als Grundlage dienen für weitere jährliche Erhebungen, um das Bewusstsein für die Gefahr des scheinbar harmlosen Feuerwerks für den Einzelnen zu schärfen.

**Zusatzmaterial online:**

Die Online-Version dieses Beitrags (10.1007/s00106-022-01259-6) enthält weiteres Zusatzmaterial: Erhebungsbogen Knalltraumregister 2021.

## Abstract (en)

**Background:**

This cross-sectional study aimed to assess the frequency and type of firework-associated acoustic trauma occurring in Germany on New Year’s Eve 2021, despite the ban on firework sales due to the COVID-19 pandemic.

**Materials and methods:**

The survey period lasted 7 days, from 28 December 2021 to 03 January 2022. A questionnaire inquired date, type and treatment of trauma, sex, and age of the patient, and whether the trauma occurred when lighting or watching fireworks. Hearing impairment was classified according to the World Health Organization (WHO grades 0 to 4), and concomitant tinnitus, vertigo, or other injuries were recorded. The questionnaire was sent to the otorhinolaryngology departments of 171 hospitals in Germany.

**Results:**

Of 37 otorhinolaryngology departments, 16 reported no and 21 reported 50 patients with firework-associated acoustic trauma. Mean age was 29 ± 16 years and 41 of 50 patients were males. Of these 50 patients, 22 presented without and 28 with hearing loss, 32 reported tinnitus and 3 vertigo; 20 patients were injured when lighting fireworks and 30 when watching. Hearing impairment was classified as 14 × WHO grade 0, 5 × WHO grade 1, 4 × WHO grade 2, 2 × WHO grade 3, and 3 × WHO grade 4. Inpatient treatment was received by 8 patients and 11 suffered from concomitant burn injuries.

**Conclusion:**

Despite the sales ban, some firework-associated acoustic traumas occurred at New Year 2021/2022 in Germany. Some instances led to hospitalization, but an even higher number of unreported cases can be assumed. This study can serve as a baseline for further annual surveys to raise the awareness of the danger of seemingly harmless fireworks for the individual.

Das heutige Feuerwerk hat seinen Ursprung in einer asiatischen Tradition, Bambus zu erhitzen, was zu typisch knallenden Geräuschen führt [8]. Das Feuerwerk soll zur Begrüßung des neuen Jahres allen Menschen Freude bringen und böse Geister vertreiben. Beginnend mit dieser Tradition um 200 vor Christus [8] hat sich seither einiges geändert: Die Leuchteffekte wurden ausgefallener und farbenfroher, der gewünschte Klangeffekt blieb.

## Ökonomische Bedeutung von Feuerwerk

Der Umsatz der pyrotechnischen Industrie in Deutschland ist in den letzten 20 Jahren annähernd kontinuierlich von 102 Mio. € im Jahr 2000 auf 137 Mio. € in den Jahren 2016 und 2017 gestiegen [18]. Dieser Umsatz erlebte zum Jahr 2020 mit dem erstmaligen Verkaufsverbot für privates Feuerwerk einen deutlichen Rückgang auf 20 Mio. € [18]. Hauptgründe für dieses Verbot waren zum einen die Reduktion von Menschenansammlungen, um die Übertragungswahrscheinlichkeit von COVID-19 zu reduzieren, zum anderen „Verletzungen beim Abbrennen von Feuerwerk in der Silvesternacht zu verhindern, um die aufgrund der Corona-Pandemie ohnehin stark beanspruchten Krankenhäuser und Notfallambulanzen zu entlasten“ [3]. Vonseiten des Verbands der pyrotechnischen Industrie wird rege Lobbyarbeit betrieben, um zukünftig zum Jahreswechsel wieder privates Feuerwerk zu ermöglichen. In einer Pressemitteilung aus dem Dezember 2021 [19] berichtet der Verband, dass von Silvesterfeuerwerk allenfalls ein geringes Schadenspotenzial für die Allgemeinbevölkerung ausgehe. So seien nur 5 % aller Krankenhausbesuche in der Silvesternacht auf Feuerwerk zurückzuführen. Darüber hinaus zitieren sie eine schriftliche Anfrage an den Bayerischen Landtag [1] hinsichtlich der Auslastung von Polizei und Rettungsdienst in der Silvesternacht 2019/2020. In Bayern seien lediglich 25 Personen durch das Abbrennen von Feuerwerk verletzt worden – eine Aussage über Alter der Betroffenen, Art und Schwere der Verletzungen fehlt jedoch, ebenso wie eine Darstellung der Situation bezogen auf die gesamte Bundesrepublik. Dem interessierten Beobachter stellt sich somit die Frage: Sind Verletzungen durch Feuerwerk an Silvester eine für das Gesundheitssystem relevante Größe mit möglicher Relevanz auch für den unbeteiligten Einzelnen? Sind sie vermeidbar und wenn ja, wie?

## Bisherige Studienlage

In mehreren Studien wurden die Schallpegel von explodierenden Feuerwerkskörpern in verschiedenen Entfernungen untersucht [8, 9, 11, 14]. So kam eine Studie zu dem Schluss, dass selbst in 6 m Entfernung vom explodierenden Feuerwerkskörper der Schallpegel über 110 Dezibel Sound Pressure Level (dB SPL,[8]) liegen kann. Je geringer der Abstand zum Feuerwerk ist, desto höher ist der messbare Schallpegel: Bei einem Abstand von 2 m wurden bis zu 160 dB SPL erreicht [14]. Dieser Wert entspricht dem Schallpegel einer abgefeuerten Pistole [13]. Professionelle Feuerwerke übersteigen diese Werte sogar mit Schallpegeln über 190 dB SPL, abhängig von der angenommenen Entfernung zur Lärmquelle [9]. Bekannt ist, dass Schallpegel von 90–130 dB SPL zur Produktion von reaktiven Sauerstoff- und Stickstoffspezies sowie weiteren freien Radikalen führen können, die die Cochlea schädigen, wohingegen Schallpegel über 130 dB SPL additiv zu einer mechanischen Schädigung des Innenohrs führen können [8]. Die akustische Wirkung von Feuerwerkslärm betrifft also nicht nur diejenigen, die das Feuerwerk aktiv zünden, sondern auch unbeteiligte Zuschauer, in der Fachliteratur „Bystander“ genannt [5, 6].

Auch für andere medizinische Fachbereiche ist das Thema von aktueller Relevanz. So hat die Deutsche Ophthalmologische Gesellschaft ein bundesweites Register zur Erfassung feuerwerksbedingter Augenverletzungen eingerichtet, um verlässliche Daten für eine Diskussion über Schutzmaßnahmen zu liefern, einschließlich eines möglichen Verbots von privatem Feuerwerk [5].

Die letzte Erhebung über akustische Traumata im Rahmen von Feuerwerk in Deutschland wurde 2002 veröffentlicht und schätzte eine Inzidenz von 10/100.000 in der Nacht zum Jahrtausendwechsel an Silvester 1999 [11]. Das deutschlandweite Verkaufsverbot für Feuerwerkskörper in den Jahren 2020 und 2021 im Zusammenhang mit der COVID-19-Pandemie wurde zum Anlass genommen, ein Register für feuerwerksbedingte akustische Traumata in Zusammenarbeit mit der Deutschen Gesellschaft für Hals-Nasen-Ohren-Heilkunde, Kopf- und Hals-Chirurgie zu erstellen – auch um der eingangs skizzierten Diskussion aus Hals-Nasen-Ohren-ärztlicher Sicht eine fundierte und aktuelle Datenbasis zu geben.

Zweck dieser Erhebungsstudie ist es, die Anzahl und die Schwere der akustischen Traumata in Zusammenhang mit Feuerwerkskörpern zu dokumentieren, die in Deutschland trotz des Verkaufsverbots für Feuerwerkskörper aufgetreten sind. Ziel ist es außerdem, die aktuell erhobenen Daten des Jahreswechsels 2021/2022 als Ausgangspunkt für weitere Erhebungen in den Folgejahren zu nutzen.

## Methoden

Diese retrospektive Querschnittstudie wurde nach den Grundsätzen der Erklärung von Helsinki durchgeführt. Sie basiert auf einem Fragebogen, der an die Abteilungen für Hals‑, Nasen‑, Ohrenheilkunde von 42 Universitätskliniken und 129 städtischen Krankenhäusern im November 2021 versandt wurde (Supplement). Die zuständige Ethikkommission bei der Ärztekammer des Saarlandes wurde informiert und entschied, dass die Studie keiner gesonderten Genehmigung bedarf, da keine personenbezogenen Patientendaten gesammelt wurden. Der Erhebungszeitraum umfasste 7 Tage um Silvester 2021 vom 28. Dezember 2021 bis zum 03. Januar 2022. Die Hals‑, Nasen- und Ohrenkliniken wurden gebeten, die Patientendaten anonym zu übermitteln und den Fragebogen zu beantworten, auch wenn sich im betreffenden Zeitraum kein Patient mit entsprechenden Kriterien vorstellte. Mittels Fragebogen wurden (1) das Datum, (2) die Art und (3) die Behandlung des akustischen Traumas abgefragt. Außerdem wurde das Geschlecht, das Patientenalter sowie die Frage berücksichtigt, ob das Trauma beim Anzünden oder Beobachten von Feuerwerk auftrat. Die Schwere der Hörbeeinträchtigung wurde – sofern eine reintonaudiometrische Testung erfolgte – nach Kriterien der Weltgesundheitsorganisation (WHO) in die Grade 0–4 eingeteilt [7, 20]. Begleitende Symptome wie Tinnitus, Schwindel sowie Begleitverletzungen wie beispielsweise Verbrennungen wurden ebenfalls erfasst. Die statistische Auswertung erfolgte deskriptiv.

## Ergebnisse

**Figure 1:**
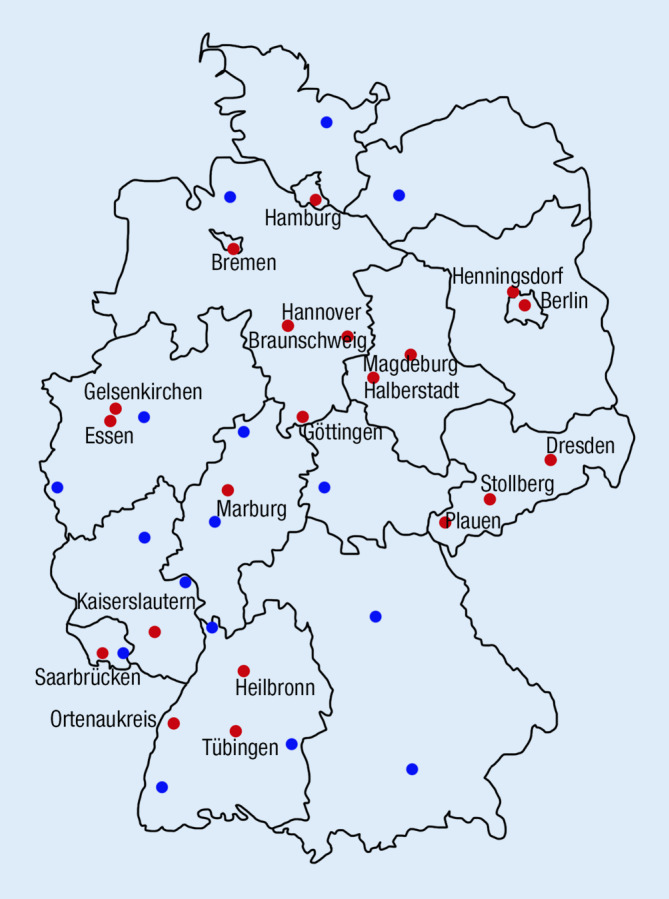

**Figure 2:**
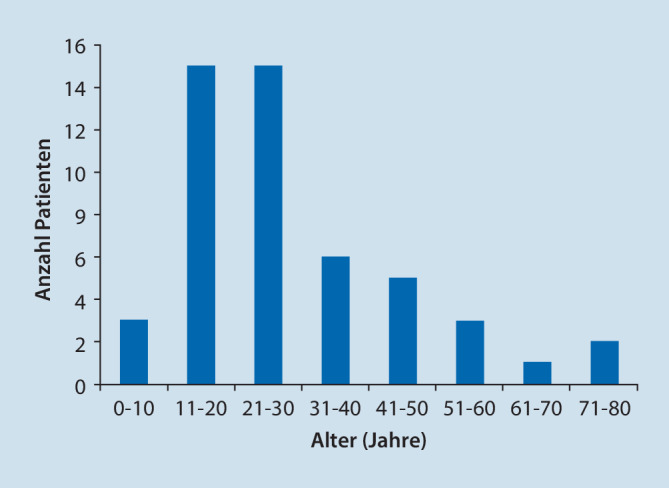

**Figure 3:**
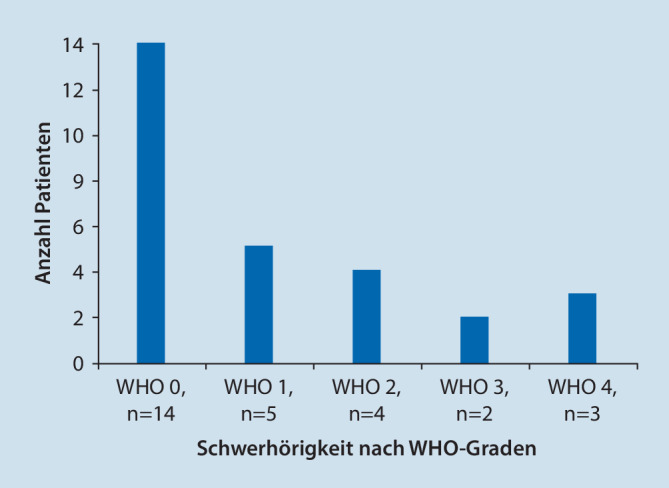

Von 171 Hals‑, Nasen-, und Ohrenkliniken stellten 37 deutschlandweit ihre Daten zu feuerwerksassoziierten akustischen Traumata zum Jahreswechsel 2021/2022 zur Verfügung. In 16 von diesen 37 Abteilungen wurden während des Erhebungszeitraums keine Patienten mit entsprechenden Charakteristika vorstellig. Die übrigen 21 Krankenhäuser meldeten insgesamt 50 Patienten mit feuerwerksassoziiertem Lärmtrauma. Die teilnehmenden Kliniken waren ohne erkennbares Muster über die gesamte Bundesrepublik verteilt (Abb. 1). Das Durchschnittsalter der gemeldeten Patienten betrug 29 ± 16 Jahre mit einer Altersspanne von 7–73 Jahren. Es waren 60 % der Betroffenen in der Alterskohorte der 11- bis 30-jährigen angesiedelt. Insgesamt 12 Betroffene waren nicht volljährig (Abb. 2). Von diesen verletzten sich 6 beim Zünden von Feuerwerk, 2 als Bystander. Bei weiteren 2 aus dieser Gruppe war der Unfallhergang unklar. Mit 41 männlichen Patienten (Durchschnittsalter 28 ± 16 Jahre, 82 %) gegenüber 9 weiblichen Patienten (Durchschnittsalter 32 ± 18 Jahre, 18 %) überwogen die Männer. Beim Anzünden von Feuerwerkskörpern selbst verletzten sich 20 Patienten (40 %), 30 (60 %) wurden als Bystander verletzt. Von den insgesamt 50 Patienten stellten sich 28 mit und 22 ohne Hörverlust vor, 32 berichteten über Tinnitus. Lediglich 3 Patienten gaben Schwindel jeweils ohne nachweisbaren Nystagmus an. Von den Patienten, die über Tinnitus oder Hörminderung berichteten, gaben 13 einen isolierten Tinnitus, 9 eine isolierte Hörminderung und 19 sowohl Hörminderung als auch Tinnitus an. Die Hörbeeinträchtigung wurde, sofern reintonaudiometrisch bestimmt, nach Kriterien der WHO als Grad 0–4 klassifiziert ([7, 20]; Abb. 3): Die Hälfte der Patienten wies einen WHO-Grad 0 auf (*n* = 14), 5 Patienten WHO-Grad 1, 4 Patienten WHO-Grad 2, 2 Patienten WHO-Grad 3 und 3 Patienten WHO-Grad 4.

Begleitverletzungen in Form von Verbrennungen meist im Gesicht und an den Händen erlitten 11 Patienten. Unter ihnen waren 2 Bystander, bei einem Patienten fehlte diese Angabe. Von den Betroffenen mit zusätzlichen Verbrennungen wurden 8 beim aktiven Anzünden von Feuerwerkskörpern verletzt, von diesen mussten 6 stationär behandelt werden.

**Figure 4:**
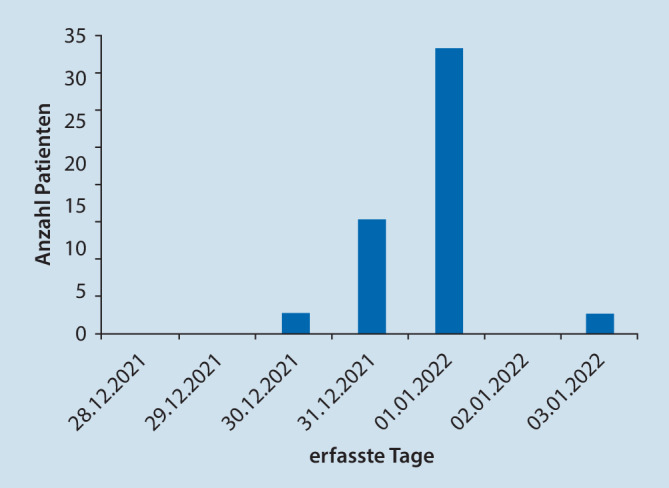

Bezüglich der zeitlichen Verteilung der gemeldeten Traumata lag das Maximum mit 33 Meldungen am 01.01.2022. Am 31.12.2021 wurden 13, am 30.12.2021 ebenso wie am 03.01.2022 wurden 2 Traumata gemeldet. (Abb. 4).

In 2 Fällen kam es zu einer neu aufgetretenen, traumatischen Trommelfellverletzung. Einer der Betroffenen hatte aufgrund einer beidseitigen chronischen Otitis media mesotympanalis bereits länger bestehende beidseitige Trommelfellperforationen.

## Diskussion

Diese retrospektive Querschnittstudie hatte das Ziel, feuerwerksbedingte akustische Traumata zum Jahreswechsel 2021/2022 in Deutschland mittels eines Fragebogens zu erfassen, der an die Hals‑, Nasen-, und Ohrenabteilungen von 42 Universitätskliniken und 129 städtischen Krankenhäusern (insgesamt 171 Krankenhäuser) gesendet wurde.

### Patientencharakteristika und betroffene Organe

Der typische Patient mit einem feuerwerksbedingten akustischen Trauma erwies sich als männlich, den Feuerwerkskörper selbst zündend und durchschnittlich 28 ± 16 Jahre alt. Insgesamt 60 % der Betroffenen waren zwischen 11 und 30 Jahre alt, 24 % hatten die Volljährigkeit noch nicht erreicht. Eine frühere Studie, die 1999 zum Millenniumswechsel in Deutschland durchgeführt wurde, ergab ebenfalls ein Überwiegen von Männern bei den Betroffenen. Akustische Traumata traten bei ihnen 3‑mal häufiger auf als bei Frauen mit einem durchschnittlichen Alter der Betroffenen von 19 Jahren [11]. Im jährlichen Feuerwerksbericht der US-amerikanischen United States Consumer Product Safety Commission von 2020 wurde festgestellt, dass das höchste Risiko für feuerwerksbedingte Verletzungen bei Personen zwischen dem 20. und 24. Lebensjahr besteht. Nach den Händen und Fingern sind die Ohren am zweithäufigsten von feuerwerksbedingten Traumata betroffen, dies zusammen mit Gesichts- und Kopfverletzungen [17]. Ähnliche Ergebnisse wurden ebenfalls in den USA publiziert durch das National Electronic Injury Surveillance System, welches von 2008 bis 2017 insgesamt 2641 feuerwerksbedingte Verletzungen erfasste. Die Ohren waren nach Verletzungen im Gesicht (62 %) sowie am übrigen Kopf (13 %) am dritthäufigsten (11 %) betroffen. Auffallend ist, dass in dieser Erhebung die Wahrscheinlichkeit einer Verletzung bei Kindern bis zum zwölften Lebensjahr am höchsten war (40 %), gefolgt von Erwachsenen über 22 Jahren (33 %) und Jugendlichen von 13–21 Jahren (27 %, [16]). Analog hierzu und zu den Zahlen von Pfisterer et al. [5] in Bezug auf Augenverletzungen sind Kinder und Jugendliche in der eigenen Studie überrepräsentiert mit 24 % gegenüber 16,7 % Anteil an der Normalbevölkerung [15]. Dabei werden Kinder und Jugendliche definiert als nicht volljährige Personen; sie betrugen 40 % in der Arbeit der Feuerwerks-Verletzungen-Studiengruppe [5].

Mit 60 % gehörte die Mehrheit der Patienten mit feuerwerksassoziiertem akustischem Trauma zur Gruppe der unbeteiligten Zuschauer. Die erhobenen Zahlen sind vergleichbar mit denen der Kollegen der Ophthalmologie [5, 6]. Eine mögliche Erklärung hierfür ist, dass sich Bystander der Gefahr durch sich möglicherweise nähernde Feuerwerkskörper weniger bewusst sind als Personen, die diese aktiv zünden, und Bystander somit keine Schutzmaßnahmen (Abstand, Gehörschutz) ergreifen können.

### Lärmschäden

Es wurde berichtet, dass Feuerwerkslärm Schalldruckpegel von bis zu 160 dB SPL erreichen kann [8, 9, 14], was dem Schalldruckpegel eines Pistolenschusses entspricht [13]. Die Annahme, dass ein adäquater Gehörschutz vor Innenohrschäden durch Schusswaffen schützt, liegt nahe, sie trifft jedoch nicht generell zu: Eine über 10 Jahre durchgeführte Längsschnittstudie bei Polizeibeamten ergab in 75 % der Fälle Veränderungen in den zervikal vestibulär evozierten myogenen Potenzialen nach monatlichen Schießübungen über 10 Jahre trotz des Tragens von Ohrschutz [21]. Die Autoren führten diese Veränderungen auf eine Schädigung durch Übertragung von Vibrationsreizen auf den Sakkulus zurück [21]. Diese Ergebnisse machen feuerwerksassoziierte akustische Traumata in zweierlei Hinsicht tückischer: Erstens kann die Kombination von Lärm und Vibration die nachteiligen Auswirkungen des Lärms auf die Cochlea verstärken [12, 21], zweitens werden Lärm und die damit verbundenen langfristigen Auswirkungen umso mehr unterschätzt, je kürzer die Expositionszeit ist. Letztgenannter Punkt ist typisch für die kurzfristige Exposition gegenüber feuerwerksbedingtem Lärm [10]. Generell gilt: Die größten absoluten Hörgewinne werden bei den am schwersten geschädigten Patienten beobachtet. Gleichzeitig sind bei diesen Patienten auch die bleibenden Schäden am größten [4].

### Vorbeugung

Zu den eingangs gestellten Fragen: Sind Verletzungen durch Feuerwerk an Silvester eine für das Gesundheitssystem relevante Größe mit möglicher Relevanz auch für den unbeteiligten Einzelnen? Sind sie vermeidbar und wenn ja, wie? Diese Fragen kann man anhand der hier zusammengetragenen Daten wie folgt beantworten: Verletzungen durch Feuerwerk sind auch für den Einzelnen relevant, treffen sie doch überproportional häufig besonders schutzbedürftige Personengruppen: Kinder und unbeteiligte Zuschauer [5, 16]. Obwohl Ersteren gemäß Sprengstoffgesetz kein Zugang zu Pyrotechnikartikeln der Kategorie 2 erlaubt ist [2], wurde die Hälfte der hier gemeldeten Kinder und Jugendlichen beim aktiven Zünden von Feuerwerk verletzt. Eine Möglichkeit, zukünftig Verletzungen zu vermeiden, ist es nach Meinung der Autoren daher, den Zugang zu Feuerwerkskörpern insbesondere für Kinder zu erschweren und auch verantwortliche Betreuungspersonen auf die konkreten Gefahren und bestehende gesetzliche Regularien hinzuweisen. Auch Zuschauer sollten für die Gefahren von Feuerwerkskörpern sensibilisiert werden.

In der vorliegenden Studie wurden Anzahl und Art von feuerwerksassoziierten akustischen Traumata in Deutschland trotz des bestehenden Verkaufsverbots für Feuerwerkskörper erhoben. Daher war eine begrenzte Anzahl an Meldungen zu erwarten. Die Autoren schließen sich der Feuerwerks-Verletzungen-Studiengruppe der Ophthalmologie an, wonach die Dynamik aufgetretener Verletzungen über die Jahre ermittelt werden sollte, um Veränderungen der Nutzung und den Effekt von gesetzlichen Regelungen – wie aktuell dem Verkaufsverbot von privatem Feuerwerk – abschließend beurteilen zu können [5].

### Limitationen der Studie

Nichtsdestotrotz bestehen jedoch auch Einschränkungen der vorliegenden Arbeit: Es wurden nur Abteilungen für Hals‑, Nasen-, und Ohrenheilkunde in Krankenhäusern kontaktiert. Möglicherweise wurden Patienten auch in Hals‑, Nasen-, und Ohren-ärztlichen Praxen behandelt und nicht an die Kliniken überwiesen. Aufgrund des Erhebungszeitraums ist es dennoch wahrscheinlich, dass die meisten Praxen geschlossen waren, was diesen möglichen Fehler zumindest abschwächt. Dennoch muss von einer relevanten Dunkelziffer ausgegangen werden, auch, weil ein Teil der Betroffenen laut Studienlage davon auszugehen scheint, dass der Hörverlust lediglich vorübergehend bestehe [11] und ein Arztbesuch nicht notwendig sei.

Eine eindeutige Einschränkung liegt sicherlich in der geringen Rücklaufquote der Meldebögen begründet, die in künftigen Erhebungen deutlich verbessert werden soll. Ein Ziel der vorliegenden Publikation ist es daher auch, das Bewusstsein für die Problematik der feuerwerksbedingten akustischen Schädigungen unter Hals‑, Nasen- und Ohrenärzten zu schärfen und so die Rücklaufquote künftiger Erhebungen zu verbessern. Ergänzend arbeiten die Autoren an der Erstellung eines Online-Fragebogens mit Eingabemodul, um perspektivisch innerhalb der nächsten 2 Jahre einen „papierfreie“ Datenerhebung zu ermöglichen.

Außerdem wurde berichtet, dass Patienten häufig das Trauma auf der Seite erleben, auf der das Feuerwerk gezündet wurde [22]. Die Autoren werden daher in künftigen Erhebungen sowohl nach der Seite der Verletzung als auch danach fragen, ob der Betroffene Rechts- oder Linkshänder ist.

## Fazit für die Praxis

- Die Anzahl an gemeldeten feuerwerksbedingten akustischen Traumata war zum Jahreswechsel 2021/2022 aufgrund des Verkaufsverbots von Feuerwerkskörpern im Rahmen der COVID-19-Pandemie gering.
- Diese Studie kann mit Einschränkungen als Grundlage für weitere jährliche Erhebungen dienen, um das Bewusstsein für die Gefahr von scheinbar harmlosen Feuerwerkskörpern für die Ohren des Einzelnen zu schärfen.
- In den kommenden Jahren sollen weitere Daten über akustische feuerwerksbedingte Traumata gesammelt werden.
- Die Auswirkungen des Verkaufsverbots von Feuerwerkskörpern im Jahr 2021 werden erst durch den Vergleich mit künftigen Erhebungen ohne Verkaufsverbot messbar und sichtbar werden.
- Geplant ist, die Umfrage erneut über den Newsletter der Deutschen Gesellschaft für Hals-Nasen-Ohren-Heilkunde, Kopf- und Hals-Chirurgie zu verbreiten.

## Supplementary Information




