# Supplementary material for: Thunder and lightning—a report on firework-associated acoustic trauma at New Year 2021/2022 (German version)
Source: HNO. 2023 Jan 5;71(1):1–7. [Article in German] doi: 10.1007/s00106-022-01259-6 (PMC9815053; doi:10.1007/s00106-022-01259-6)
Supplement: Supplementary file 1 [file 106_2022_1259_MOESM1_ESM.pdf]

# Erhebungsbogen Knalltraumaregister 2021

| | | |
|---|---|---|
| Unfalltag<br>(Datum) | | Selbst gezündet ☐<br>oder Zuschauer ☐ ? |
| Alter | Jahre | |
| Geschlecht | M: W: | |

***Art der Verletzung***

| | | | | | |
|---|---|---|---|---|---|
| **Hörschaden** | Nein | Ja | | | |
| | | Sensorineural | Schallleitung | kombiniert | |
| | | WHO-Grad<br>Mittlerer Hörverlust aus dem Mittelwert im Reintonaudiogramm bei 500, 1000, 2000, 4000 Hz | 0 | 25dB oder besser | |
| | | | 1 | 26-40db | |
| | | | 2 | 41-60db | |
| | | | 3 | 61-80db | |
| | | | 4 | 81db oder mehr | |
| | | Behandlung | | | |
| **Tinnitus** | Nein | Ja | | | |
| **Schwindel** | Nein | Ja | | | |
| | | Spontan-<br>nystagmus? | Nein | Ja | |
| | | | | rechts / links / vertikal | |
| **Begleit-<br>verletzungen** | Nein | Ja | | | |
| | | Trommelfell-<br>verletzung: | Nein | Ja | |

***Art der Behandlung***

| **Ambulant** | **Stationär** |
|---|---|
| Medikamentös (z.B. Cortison, Pentoxifyllin, Tebonin…) | Operativ (Infusion, Trommelfellschienung, Myringoplastik, Rundfensterabdeckung…) |

***Bemerkungen/Ergänzungen***

| |
|---|
| |

Klinikstempel

Name Behandler

**Bitte senden an**:
**FAX**: +4968411622997
**Email**: knalltrauma@uks.eu
**Post**:
Prof. Dr. med. A. Bozzato
Klinik für Hals-, Nasen- und Ohrenheilkunde
UKS – Universitätsklinikum des Saarlandes
Gebäude 6
D-66421 Homburg
